# Trimmer's Cottage Hospital, Farnham

**Published:** 1896-02-29

**Authors:** 


### EDITOR'S LETTER-BOX.

[Our correspondents are reminded that proxility is a great bar to publication, and that brevity of style and conciseness of statement greatly facilitate early insertion.]

### TRIMMER'S COTTAGE HOSPITAL, FARNHAM.

MR. ALFRED EGGAR, the architect of the above hospital, writes: Referring to the remarks in THE HOSPITAL on this building, I wish to point out that the bathroom has direct communication with the open air over the glazed corridor, the window being the entire width of the room, 1 ft. 6 in. deep, and hung on joists. I enclose plan showing the approach to operating-room. The corridor has glazed doors, which open right back in case of need, so that a stretcher can be carried in on the level practically through only one door. I hope you will call attention to this in your next issue.

*** Mr. Eggar explains that the bath-room has direct communication with the outer air above the roof of the glazed corridor. This is a more satisfactory arrangement than the plan appears to indicate, apart from the explanation; but, if it is sufficiently lighted and ventilated thus, the window shown opening into the corridor appears superfluous, and calculated to deprive the bath-room of privacy. The entrance to the operation-room is criticised as objectionable for patients brought from the wards, and the access to it from these is obviously exceedingly awkward. Mr. Eggar explains the approach as though patients were brought in only from the "hall and waiting-room," but it is sometimes necessary to operate on ward patients, and the criticism would then probably find its justification.—ED. *T.H.*

